# Comprehensive virome analysis of *Varroa destructor* populations in South Korea

**DOI:** 10.3389/finsc.2026.1757017

**Published:** 2026-01-28

**Authors:** Ji-Young Kim, Kyung Hwan Moon, Young Ho Kim, Nurit Eliash, June-Sun Yoon

**Affiliations:** 1 Insect Physiology and Molecular Biology Laboratory, Department of Agricultural Convergence Technology, Jeonbuk National University, Jeonju, Republic of Korea; 2 Department of Vector Entomology, Kyungpook National University, Sangju, Republic of Korea; 3 Shamir Research Institute, Katzrin, Israel; 4 University of Haifa, Haifa, Israel

**Keywords:** high-throughput sequencing, honey bee, varroa destructor, virome analysis, viruses

## Abstract

The ectoparasitic mite *Varroa destructor* and the viruses it transmits pose a significant threat to honey bee health (*Apis mellifera*), contributing to colony collapse disorder. In this study, a metatranscriptomic analysis of *V. destructor* from six regions in South Korea was conducted to characterize its viral communities. Using high-throughput sequencing (HTS), we identified 16 known viruses and classified them into three groups: honey bee-pathogenic, Varroa destructor viruses (VDVs), and viruses with unknown hosts. Reverse transcription polymerase chain reaction (RT-PCR) was used to validate the HTS results, revealing only two discrepancies out of 96 comparisons. This emphasizes the importance of integrating both methods for comprehensive virome analysis. Deformed wing virus was the most prevalent and abundant virus, comprising 75%–99% of viral reads in five out of six farms. One farm showed a high abundance of VDVs (3 and 9). Notably, two previously unreported viruses with unknown hosts, Hubei partiti-like virus 34 and Lilac leaf chlorosis virus (LLCV), were identified. For LLCV, the detection of all ribonucleic acid segments highlighted the critical impact of sequencing depth on viral genome analysis. To our knowledge, this study provides the first virome characterization of *V. destructor* in South Korea, revealing diverse viral communities. It also proposes an integrated analytical approach using RT-PCR and HTS, emphasizing the importance of sequencing depth. This analysis provides valuable insights into the potential impacts of viral infections on honey bee colony health and the epidemiology of viral transmission.

## 1 Introduction

*Apis mellifera* is a globally significant pollinator that plays a vital role in agricultural ecosystems by pollinating food crops and wild plants (1). In recent years, there has been a decline in honey bee populations due to the growing crisis of colony loss (2, 3). These declines are attributed to a combination of biotic and abiotic factors (4), with *Varroa destructor* and the viruses it transmits playing major roles in colony loss (5, 6). *V. destructor* parasitizes honey bee pupae by penetrating feeding holes in the abdominal sternites and extracting hemolymph and fat body, thereby compromising the host’s humoral immune system (7). As a result, the honey bee’s immune system is suppressed, making them highly susceptible to viral infections (8). Moreover, *V. destructor* serves as a vector for pathogenic viruses that are transmitted to honey bees during feeding (9). Hence, when *Varroa* mites are present in colonies, they can contribute to colony loss by impairing the host immune system and facilitating the transmission of viruses (6).

*V. destructor* mediates a variety of viruses, including pathogenic viruses in honey bees. Currently, more than 20 viral species have been identified in *V. destructor* (10–12), among which deformed wing virus (DWV), sacbrood virus (SBV), Israeli acute paralysis virus (IAPV), acute bee paralysis virus (ABPV), black queen cell virus (BQCV), Kashmir bee virus (KBV), and chronic bee paralysis virus (CBPV) cause severe diseases in honey bees (13). DWV replicates within the *Varroa* mite and suppresses host immunity, resulting in higher levels of DWV transmitted to honey bees (5, 12). DWV causes developmental deformities and premature aging, leading to high overwintering colony losses (14–16) ABPV, KBV, and IAPV (the AKI complex) belong to the same genus, *Aparavirus* (family *Dicistroviridae*), and share genome similarity (17). *V. destructor* serves as an effective vector of the AKI complex and causes acute paralysis in honey bees (18). BQCV and SBV primarily infect honey bees, causing failure in pupation and persisting in adult honey bees as asymptomatic infection (19–21). CBPV causes a severe chronic paralytic disease in adult honey bees (22). Several RNA viruses specific to *Varroa* (*Varroa destructor* virus 2, *Varroa destructor* virus 3, *Varroa destructor* virus 4, *Varroa destructor* virus 5, and *Varroa destructor* virus 9) have been identified in this ectoparasitic mite (23–25). These viruses can replicate not only in *V. destructor* but also in *A. mellifera*, indicating that their presence in honey bees is due to the *Varroa*’s feeding behavior. Most viruses found in *V. destructor* belong to positive-sense single-stranded RNA viruses, although a few other viral types have also been identified. Negative-sense RNA viruses, such as Apis rhabdovirus 1 (ARV1) and Apis rhabdovirus 2 (ARV2), and double-stranded DNA viruses, such as *Apis mellifera* filamentous virus, have also been identified in *Varroa* mites (26, 27).

Previous research on viruses associated with *V. destructor* in South Korea was conducted by Moon et al. (28), who identified six honey bee pathogenic viruses (DWV, SBV, IAPV, BQCV, CBPV, and KBV) in *V. destructor* from 46 apiaries across the country using the PCR method, with DWV showing the highest prevalence. However, PCR-based methods using specific primers have certain limitations in detecting novel viruses and quantifying viral loads (29).

Over the past decade, high-throughput sequencing (HTS) has revolutionized virology by enabling the identification of viral abundance in hosts (30). This approach allows for the detection of novel viruses and has significantly shifted the paradigm of viral diversity and ecological dynamics in insects such as mosquitoes and bees (31–33). In parallel, several countries have conducted mass screenings to explore potential virological links between *V. destructor* and colony collapse disorder (CCD) (10, 11, 19, 24, 34). For example, Lester et al. (2022) performed RNA-seq analysis of *V. destructor* collected across New Zealand and identified 10 viruses, including nine that were also found in honey bees (34). Similarly, Levin et al. (2016) reported 22 viruses in *V. destructor*, including two that had not been previously described (25). A recent study from China reported the discovery of more than 23 novel viruses in V. destructor and A. mellifera colonies, with the viral community in V. destructor exhibiting greater richness compared to that in *A. mellifera* (19). In addition, Eliash et al. (2022) analyzed a large dataset of 66 *Varroa* transcriptomes and identified at least three types of viruses, including VDV2, which was co-infecting with *V. destructor* (11). Virome analysis using HTS allows for consistent scale studies of the spatial and temporal distribution of viral communities and facilitates the discovery of novel pathogenic viruses within *V. destructor* (35).

Currently, *Varroa* mite populations in South Korea have developed resistance to several pesticides, including pyrethroids and formamidines (36). This resistance results in failure to control *Varroa* mites, threatening conventional *Varroa* management strategies for maintaining healthy honey bee colonies. Therefore, the ectoparasitic mite *V. destructor* has been recognized as the primary cause of honey bee population losses during overwintering in South Korea (37). Moreover, previous research on *Varroa*-associated viruses has primarily focused on detecting known pathogenic viruses using PCR assays (28). Therefore, in this study, we conducted a comprehensive virome analysis of *V. destructor* using the HTS approach. Metatranscriptomic data were obtained from mites collected across six geographically distinct regions of South Korea in 2022, which enabled the identification of the composition, distribution, and abundance of the viral community. The distribution and abundance of the virus were found to be different among the six regions. These findings were further validated using PCR-based assays. We also conducted phylogenetic analyses to investigate the genetic relationships among viral genotypes and infer their possible evolutionary origins. Our study provides a comprehensive overview of the viral community within *V. destructor* populations in South Korea and also fundamental insights into the dynamics of *Varroa*-mediated viral transmission.

## 2 Materials and methods

### 2.1 Insects and RNA sequencing

*V. destructor* samples were collected from multiple hives across six regions in South Korea in September 2022 and were placed in 70% ethanol and stored at −80°C. These samples were pooled into groups of 10–20 individuals per sample (**Supplementary Data 1** in **Supplementary File 1**) and transferred to 2 mL tubes containing Total RNA Isolation Reagent^®^ (Molecular Research Center, Cincinnati, OH, USA) along with four steel beads. Each pooled sample was thoroughly homogenized using a Precellys Evolution (Bertin Technologies, Montigny-le-Bretonneux, France) at 6,300 rpm for two cycles of 20 s each, with a 30-s pause. After homogenization, RNA extraction was performed using the Direct-zol™ RNA MiniPrep Plus (Zymo Research, Irvine, CA, USA), following the manufacturer’s instructions. DNase I (Thermo Fisher Scientific, Waltham, MA, USA) was added during RNA extraction to remove residual DNA. The concentration and purity of the RNA were evaluated using a QuickDrop spectrophotometer (Molecular Devices, Sunnyvale, CA, USA), and the samples were stored at −80 °C.

Total RNA concentration was calculated using Quant-IT RiboGreen (Invitrogen, Waltham, MA, USA). To evaluate the integrity of the total RNA, the samples were run on TapeStation RNA ScreenTape (Agilent Technologies, Santa Clara, CA, USA), and only high-quality RNA samples with an RIN value greater than 5.0 were used for constructing RNA libraries. An RNA library was prepared using 0.5 µg of total RNA for each sample using Illumina TruSeq Stranded Total RNA Library Prep Gold Kit (Illumina, Inc., San Diego, CA, USA). After the removal of rRNA, the remaining mRNA was fragmented into small pieces using divalent cations under temperature. The cleaved RNA fragments were copied into first-strand cDNA using SuperScript II reverse transcriptase (Invitrogen, Waltham, MA, USA) and random primers, followed by second-strand cDNA synthesis using DNA polymerase I, RNase H, and dUTP. These cDNA fragments then underwent an end-repair process, addition of a single “A” base, and then ligation of the adapters. The products were then purified and enriched by PCR to generate the final cDNA library. The libraries were quantified using KAPA Library Quantification kits for Illumina Sequencing platforms according to the qPCR Quantification Protocol Guide (KAPA BIOSYSTEMS, Wilmington, MA, USA) and qualified using TapeStation D1000 ScreenTape (Agilent Technologies, Santa Clara, CA, USA). Indexed libraries were then submitted to Illumina NovaSeqX (Illumina, Inc., San Diego, CA, USA), and paired-end (2 × 150 bp) sequencing was performed by Macrogen Incorporated (Macrogen, Seoul, South Korea). The sequencing depth was calculated using the total number of reads per sample. All samples were sequenced at a 40× depth. For farm 2, we requested 90× depth to capture the sequencing accuracy.

### 2.2 Virome analysis

Virome analysis was conducted using paired-end sequencing data derived from the Illumina platform. Quality control of the raw reads was performed using FastQC (version 0.12.1) (38). Adapters and low-quality reads (Phred quality score <30) were removed using FastP (version 0.23.4) (39). The clean reads were aligned to the host genome assembly of *V. destructor* (accession: GCA000181155.2). Host-derived reads were removed by mapping them to the host genome using Bowtie2 (version 2.5.4) (40). Unmapped reads were then assembled using metaviralSPAdes (version 4.0.0) (41). To identify viral transcripts, the assembled contigs were aligned against the NCBI RefSeq database using the BLASTn algorithm. Viral genomes were confirmed using a cutoff E-value of 1e-50. Taxonomic classification of the viruses was determined using the NCBI reference genome database. The abundance of viral transcripts was quantified using the transcripts per million (TPM) value with Kallisto (version 0.46.2) (42).

### 2.3 Phylogenetic analysis

A phylogenetic analysis was performed for the following viruses: DWV-A, SBV, and ARV1. Their sequences were aligned using ClustalW (version 2.0) (43), and a phylogenetic tree was constructed using the maximum likelihood method in the MEGA11 program (version 11.0.10) (44). The phylogenetic relationships were displayed as a rooted phylogenetic tree. This branch was determined by performing 1,000 bootstrap replications.

### 2.4 RT-PCR assays

RT-PCR was performed to confirm the presence of 16 viruses in *V. destructor* identified through transcriptomic analysis. After sequencing, the remaining RNA (total 500 ng) was used to generate cDNA using ReverTra Ace qPCR RT Master Mix with gDNA Remover (Toyobo, Osaka, Japan), following the manufacturer’s protocol. Primer sets were designed using the PrimerQuest Tool provided by Integrated DNA Technologies (https://sg.idtdna.com/pages/tools/primerquest). The RT-PCR mixture consisted of 10 µL AccuPower Master Mix (Bioneer, Daejeon, South Korea), 1 µL of each primer (10 µM), and 1 µL of cDNA, prepared to a total volume of 8 µL with nuclease-free water. The PCR cycling conditions were as follows: 95 °C for 5 min, followed by 35 cycles of 95 °C for 15 s, 55 °C for 30 s, and 72 °C for 60 s, with a final extension at 72 °C for 5 min. The PCR products were separated by 1% agarose gel electrophoresis and visualized using SYBR™ Green I Nucleic Acid Gel Stain (Invitrogen, Waltham, MA, USA).

## 3 Results

### 3.1 Viral community of *V. destructor*

**Figure 1:**
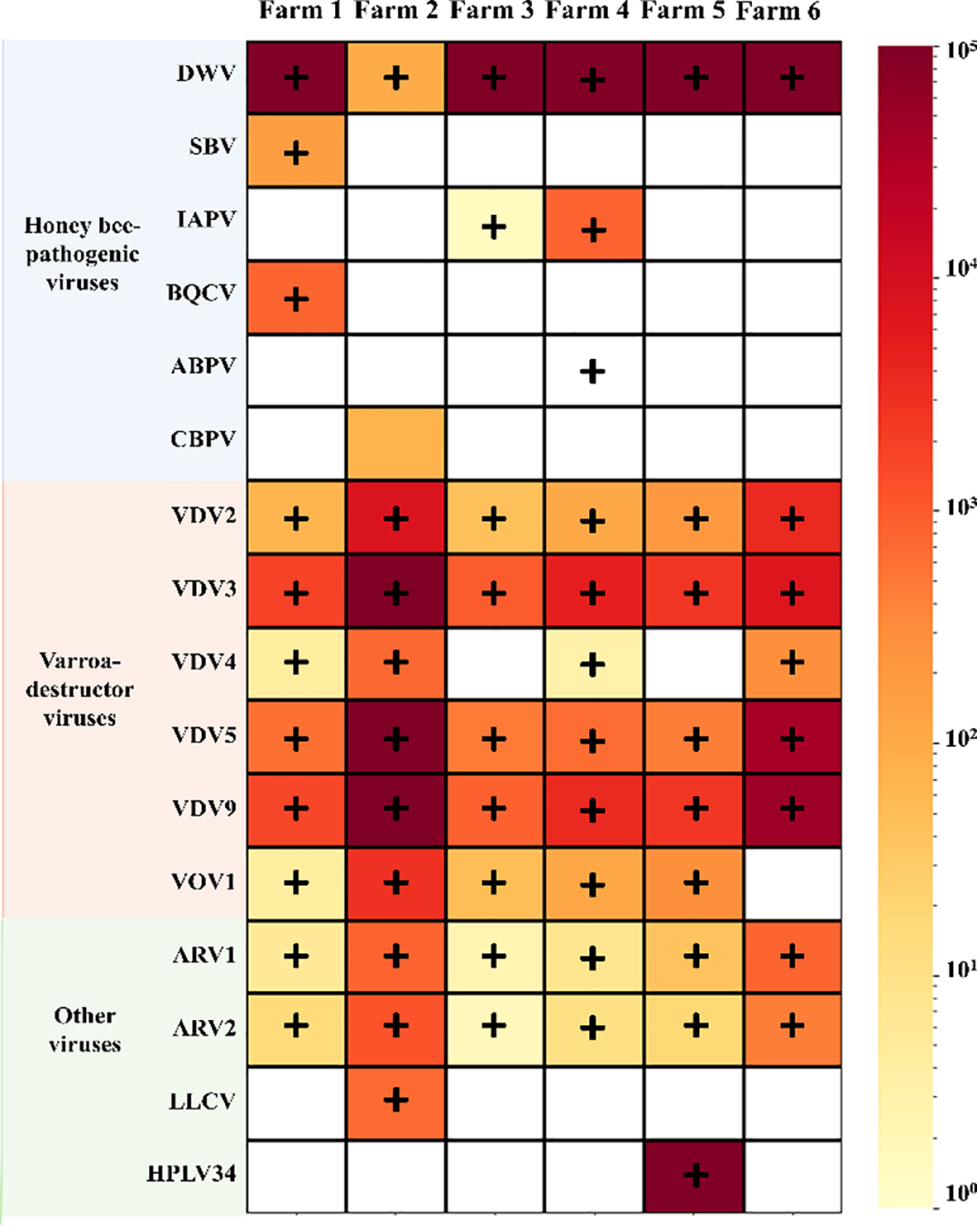
Heatmap of the distribution and abundance of 16 viruses in the six samples. The Y-axis represents the 16 identified viruses in this study, grouped by their primary host (honey bee–pathogenic viruses, Varroa destructor viruses, and other viruses). The X-axis represents the six different farms where *V. destructor* samples were collected. The heatmap represents the relative abundance of virus-based TPM values, according to the color gradient legend in the right panel. The “+” mark indicates the sample that was confirmed by PCR. The white boxes indicate that no viral reads were detected in the corresponding samples.

Our study demonstrated the viral distribution and abundance of *V. destructor* in six different farms across South Korea using HTS (**Supplementary Data 1** in **Supplementary File 1**). Of the 26 viruses known to be infected by *V. destructor*, 16 were identified in at least one of the six samples (**Figure 1**). The viruses were classified into three groups based on their major host: honey bee pathogenic viruses, *Varroa destructor* viruses, and other viruses with unknown hosts. The first group comprised six honey bee pathogenic viruses: DWV, SBV, IAPV, BQCV, ABPV, and CBPV. The second group included viruses with *V. destructor* as the primary host, namely VDV2, VDV3, VDV4, VDV5, VDV9, and *V*arroa orthomyxovirus 1 (VOV1). The third group comprised ARV1, ARV2, Lilac leaf chlorosis virus (LLCV), and Hubei partiti-like virus 34 (HPLV34), whose primary hosts remain unidentified.

### 3.2 Overview of viruses detected in *V. destructor* from South Korea

#### 3.2.1 Distribution of viruses across six farms

We examined the presence of 16 viruses in *V. destructor* samples collected from six farms across South Korea. Each sample was found to be coinfected with at least eight viruses. Among honey bee-pathogenic viruses, DWV was detected in all six regions, and IAPV was identified in samples collected from farms 3 and 4. BQCV was exclusively detected in farm 1. ABPV and CBPV were detected in farms 2 and 4. V*. destructor* viruses, including VDV2, VDV3, VDV5, and VDV9, were consistently identified in all samples, whereas VDV4 was not detected in samples collected from farms 3 and 5. VOV1 was identified in all samples except in samples collected from farm 6. ARV1 and ARV2 were also detected in all samples. LLCV and HPLV34 were only identified in farms 2 and 5 (**Figure 1**). We performed RT-PCR to validate the HTS results, which revealed two discrepancies of 96 RT-PCR assays: CBPV and ABPV. CBPV was detected by HTS in farm 2 but not by PCR. In contrast, ABPV was detected by PCR but not by HTS. HTS and RT-PCR exhibited a low overall error rate of 0.01%.

#### 3.2.2 Abundance of viruses across six farms

**Figure 2:**
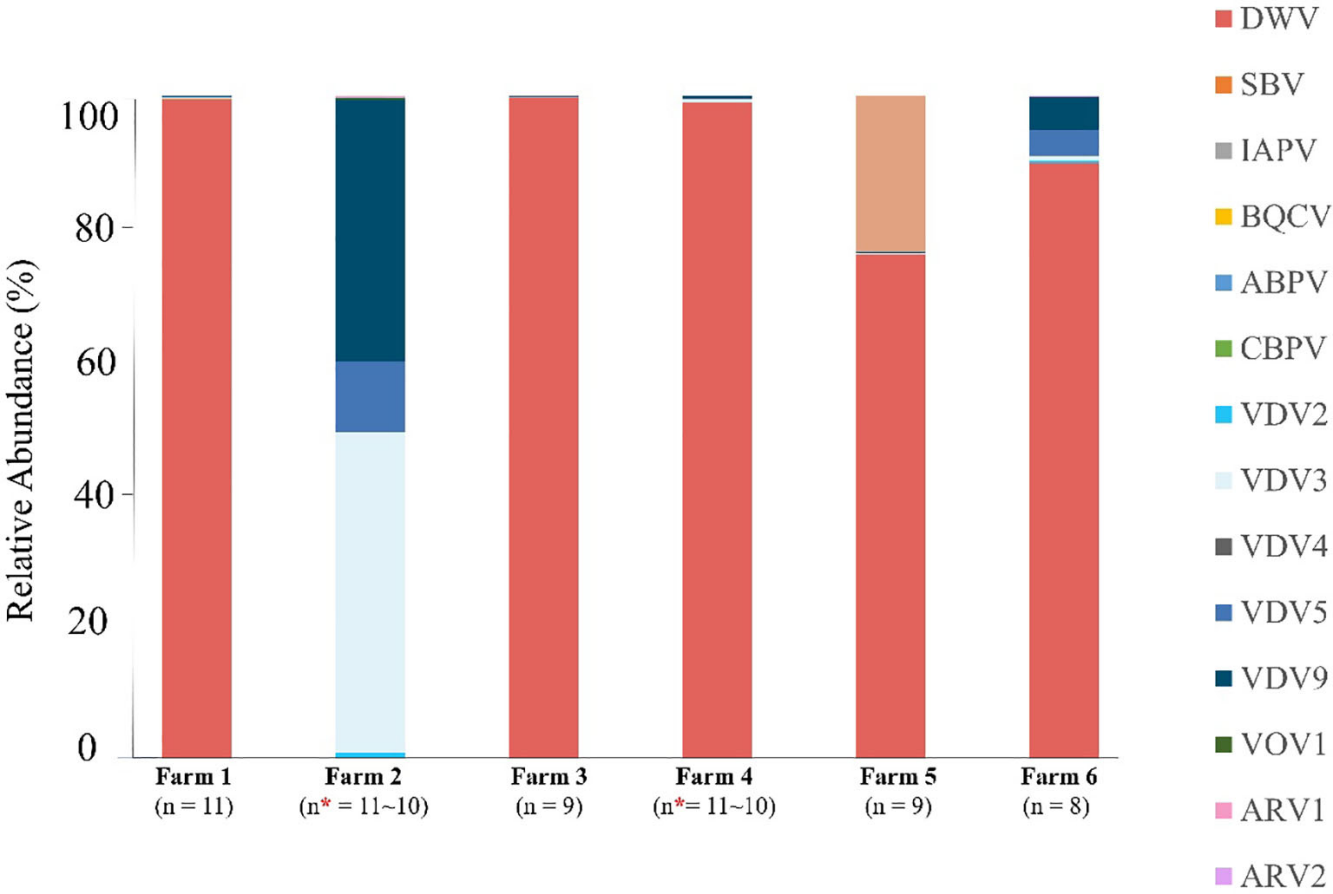
Viral compositions of *V. destructor* from the six samples. Relative abundance (%) of viruses detected in *Varroa* mites from six farms. Each bar represents the viral composition of a single mite sample, with colors indicating the individual virus species. N = total number of co-infecting viruses detected per farm. (*) indicates discrepancies between RT-PCR and HTS results.

Analysis of the relative abundance of viruses revealed DWV as the predominant virus in five farms, whereas farm 2 displayed a different pattern of viral abundance (**Figure 2**).

#### 3.2.3 DWV-predominant samples: Farms 1, 3, 4, 5, and 6

DWV was the most abundant virus in samples collected from farms 1, 3, 4, 5, and 6, accounting for 75%–99% of the total viral reads. In three farms (1, 3, and 4), DWV accounted for 98%~99% of the total viral reads. The proportion of other viruses did not exceed >1% of the total viral reads. In farm 5, DWV accounted for 74%, with HPLV34 comprising for 23% of the total viral reads. Consistently, DWV was the most abundant virus in farm 6, accounting for 89%, followed by VDV9 at 9%.

#### 3.2.4 DWV-low abundance sample: Farm 2

DWV exhibited high levels of abundance in most samples, whereas farm 2 demonstrated relatively low abundance. In farm 2, DWV accounted for 1% of the total viral reads. In contrast, VDV3 was the most abundant virus in farm 2, accounting for 48% of the total viral reads, followed by VDV9, which accounted for 39%.

### 3.3 Phylogenetic tree of three viruses (DWV, ARV1, and ARV2)

**Figure 3:**
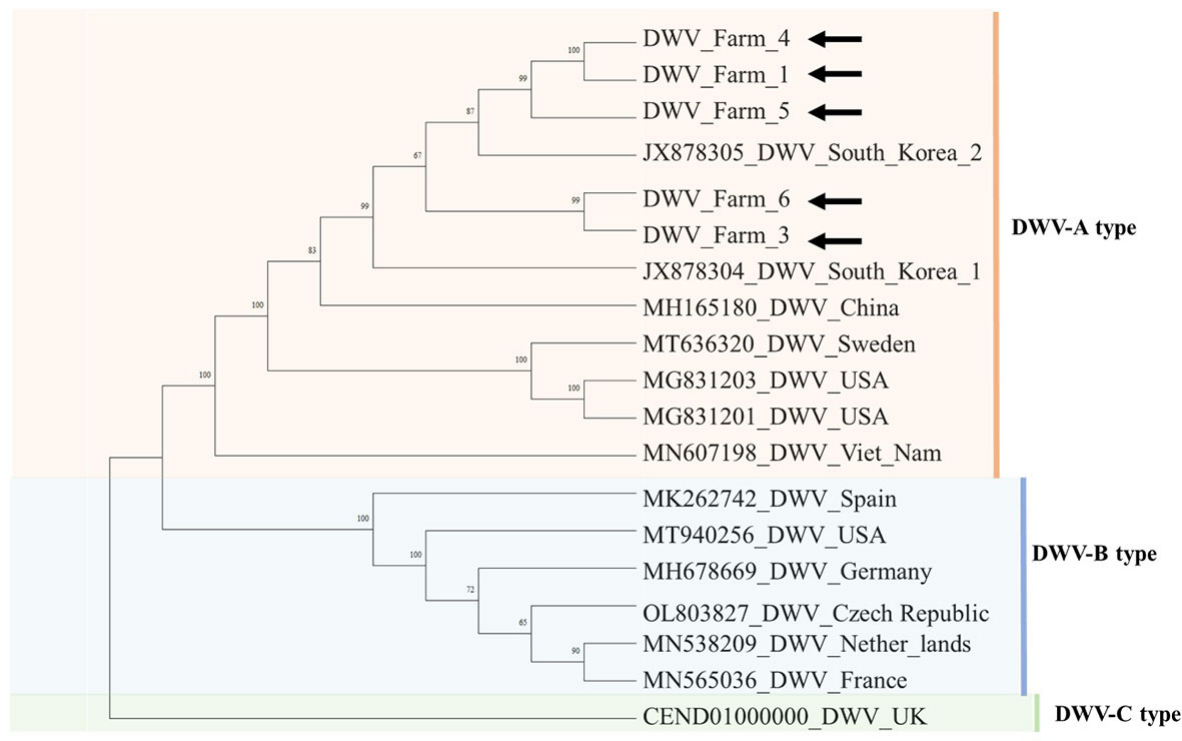
Phylogenetic tree of DWV. The DWV sequences were aligned using ClustalW. The phylogenetic tree was constructed using the neighbor-joining method, with bootstrap values based on 1,000 replicates. Samples from this study are indicated by black arrows.

Phylogenetic analyses of three viruses (DWV, ARV1, and ARV2) whose genotypes and regional strains were associated with virulence revealed distinct phylogenetic groups according to the genetic differences among the viruses (**Figure 3**).

#### 3.3.1 Deformed wing virus

Five DWV sequences were obtained in this study, and the similarity between sequences was confirmed as 96%~97%. Phylogenetic analysis revealed that all five sequences were clustered in the DWV-A clade and were closely related to the DWV strain Korea-2 (JX878305) (**Figure 3**). Results showed that the DWV-A clade predominantly comprised isolates from Central and Southeast Asian countries, such as China and Vietnam. DWV-B and DWV-C were primarily detected in Europe and the United States. These results demonstrated that DWV-A is the predominant genotype in South Korea.

#### 3.3.2 Sacbrood virus

**Figure 4:**
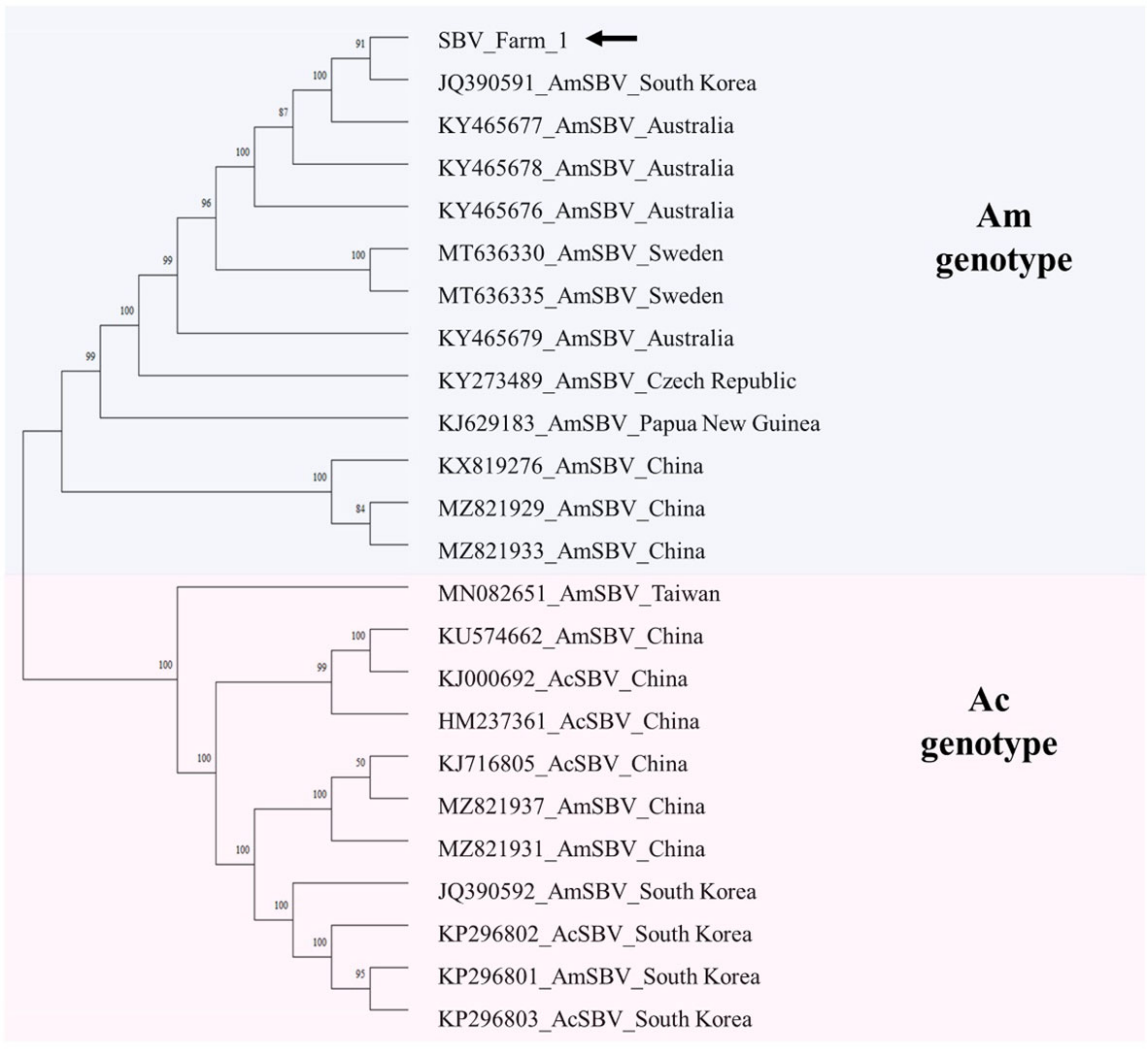
Phylogenetic tree of SBV. The SBV sequences were aligned using ClustalW. The phylogenetic tree was constructed using the neighbor-joining method, with bootstrap values based on 1,000 replicates. Samples from this study are indicated by black arrows.

Phylogenetic analysis was conducted based on SBV identified in farm 1. The phylogenetic tree clearly diverged into two main branches, corresponding to host species, specifically *A. mellifera* genotype (Am) and *A. cerana* genotype (Ac). The SBV isolates from *A. mellifera* comprised the Am genotype, whereas those from *A. cerana* comprised Am and Ac genotypes. Phylogenetic analysis revealed that SBV detected in this study was classified as the Am genotype and was closely related to SBV (JQ390591) isolated from *A. mellifera* in South Korea (**Figure 4**).

#### 3.3.3 Apis rhabdovirus

**Figure 5:**
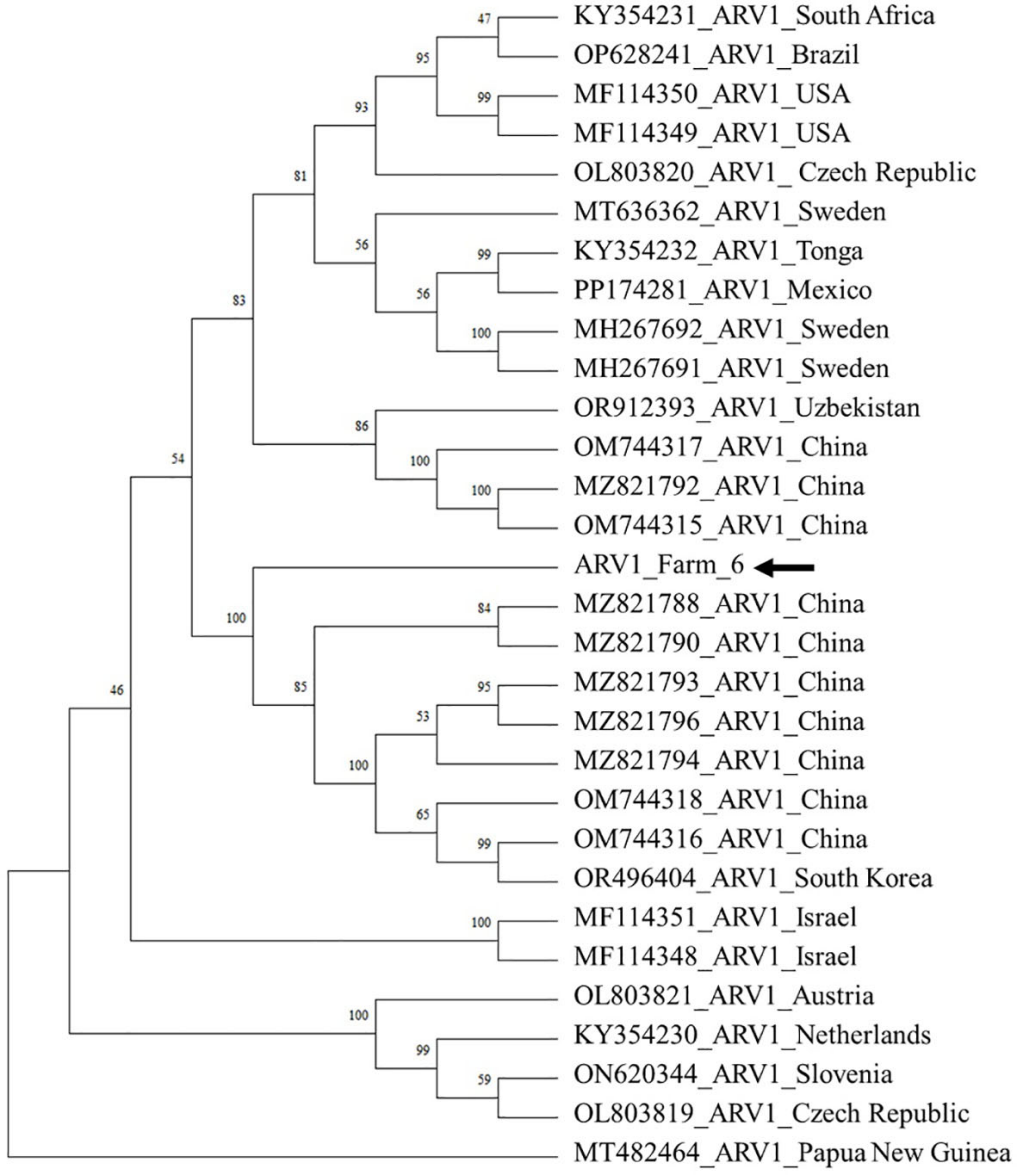
Phylogenetic tree of ARV1. The ARV1 sequences were aligned using ClustalW. The phylogenetic tree was constructed using the neighbor-joining method, with bootstrap values based on 1,000 replicates. Samples from this study are highlighted using a black arrow.

We compared ARV1 with 30 genomes available in GenBank to evaluate its regional characteristics. The genome of ARV1 obtained from farm 6 demonstrated high sequence similarity with an ARV1 isolate from China (MZ821788) (**Figure 5**). The phylogenetic analysis revealed that ARV1 demonstrated 99.4% similarity to *A. mellifera* isolated from South Korea (OR496404). These results suggest that the Korean ARV1 is genetically closely related to strains from China.

### 3.4 Identification of previously unreported viruses in *V. destructor*

**Figure 6:**
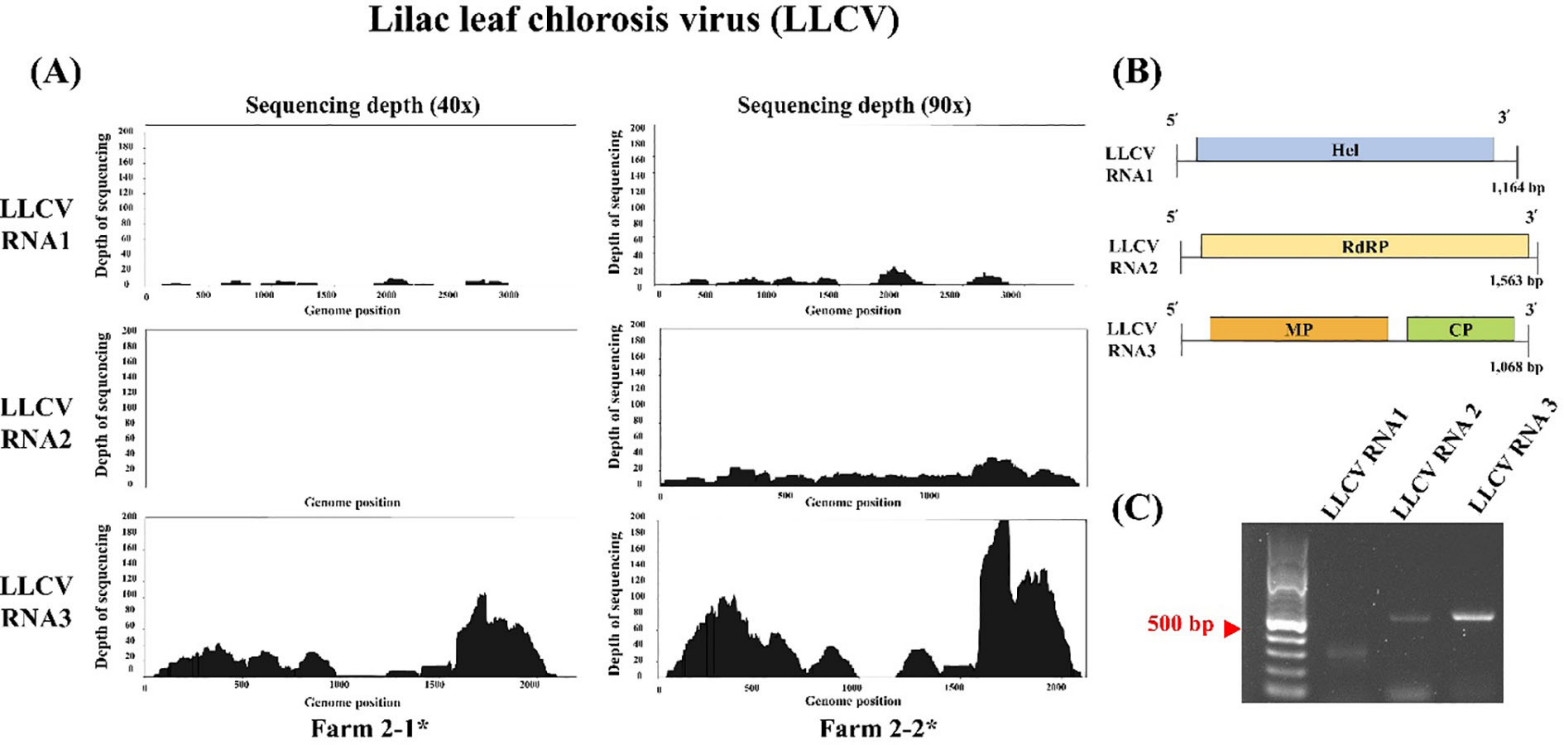
Previously unreported LLCVs in V. destructor identified in this study. **(A)** Mapping coverage plot based on read mapping of the Lilac leaf chlorosis virus. Sequencing reads were mapped to the Lilac leaf chlorosis virus reference genome. The x-axis represents the genome position (bp), and the y-axis represents the sequencing depth (reads). The left panel shows coverage generated through 40× sequencing; the right panel shows coverage at a sequencing depth of 90×. **(B)** Genome of Lilac leaf chlorosis virus in *V. destructor*. The genome structure of the virus comprises three RNA segments: RNA1, RNA2, and RNA3. RNA1 encodes a helicase protein; RNA2 encodes a polyprotein including RNA-dependent RNA polymerase (RdRP); and RNA3 encodes two proteins, movement protein (MP) and coat protein (CP). **(C)** RT-PCR analysis of Lilac leaf chlorosis virus. Gel electrophoresis results confirmed the presence of RNA1, RNA2, and RNA3.

The HTS analysis identified two viruses that had not been previously reported in *V. destructor*: LLCV in farm 2 and HPLV34 sequence in farm 5. HPLV34 had a genome length of 1,022 nt and contained a single open reading frame (ORF) (**Supplementary Data 5A** in **Supplementary File 1**). This ORF exhibited 99.67% nucleotide sequence similarity to the RNA-dependent RNA polymerase (RdRp) of HPLV34 (ON648754). RT-PCR was performed to validate the presence of virus in farm 5 (**Supplementary Data 5B** in **Supplementary File 1**). Furthermore, LLCV contains three RNA segments (RNA1, RNA2, and RNA3) (**Figure 4**). At 40× sequencing depth, the viral segments LLCV RNA1 and RNA3 were detected, and BLAST analysis revealed 88.67% similarity to LLCV RNA1 (NC025477) and 95% similarity to LLCV RNA3 (NC025481) (**Figure 6A**). To confirm the presence of RNA2, the same sample was analyzed at 90× sequencing depth, where the LLCV RNA2 sequence was identified (**Figure 6A**). BLAST hits also demonstrated 95% similarity to LLCV RNA2 (NC025478). RNA1 was predicted to encode helicase (Hel), and RNA2 was predicted to encode RdRp. RNA3 was identified as containing two ORFs, including the movement protein (MP) and the coat protein (CP) (**Figure 6B**). We performed RT-PCR to validate the presence of all three segments of LLCV, which were detected in farm 2 (**Figure 6C**). Hence, in this study, the genomes of HPLV34 and LLCV were identified, which had not been previously reported in *V. destructor*, and their presence was confirmed using RT-PCR.

## 4 Discussion

This study conducted a comprehensive virome analysis of *V. destructor*, an ectoparasitic mite of honey bees. *Varroa* mites and viruses transmitted by *V. destructor* are the major causes of CCD. Of the 26 viruses known to infect *V. destructor* (11, 24), 16 were identified in our HTS data. The viruses identified from *Varroa* mites were classified into three groups: honey bee-pathogenic viruses, *Varroa destructor* viruses, and other viruses. This study described the viral community of *V. destructor* collected in six regions of South Korea in 2022 and emphasized the significant differences in viral composition and distribution across the regions, thereby contributing to our understanding of the viral epidemiology of *V. destructor*.

The seven viruses: DWV, SBV, IAPV, BQCV, ABPV, CBPV, and KBV, are pathogenic to *A. mellifera* (13, 21, 45–47). Of the seven honey bee–pathogenic viruses, six were detected in our samples, with KBV absent from all regions. *DWV was detected in all six regions, whereas the other viruses were detected in only one or two regions; therefore, the geographic distribution of DWV was compared with that of the other viruses.* DWV, the most prevalent virus in global beekeeping, exhibits increasing prevalence when the ectoparasite *V. destructor* is present within colonies (48, 49). The abundance of DWV accounted for 76%–99% of the total viral reads in all farms, except farm 2, where its relative abundance was low. DWV has been classified into three primary genotypes: DWV-A, DWV-B, and DWV-C, among which DWV-A was the most prevalent (50, 51). In contrast, the DWV-B genotype is highly pathogenic and has recently become dominant in the United States and Europe (52). According to phylogenetic analysis, the DWVs identified in our samples were categorized as DWV-A (**Figure 3**). This result indicated DWV-A as the predominant genotype of *V. destructor* in South Korea in 2022. This finding is consistent with previous studies that highlighted DWV-A as the most prevalent genotype in East Asia, including South Korea (50, 52). Among the six regions, SBV was detected only in farm 1. SBV was classified into two types: AmSBV, which infects only western honey bees (*A. mellifera*), and AcSBV, which infects both western honey bees (*A. mellifera*) and eastern honey bees (*A. cerana*) (53, 54). According to our genome analysis, the SBV detected in our study was the AmSBV type, consistent with previous research that reported an identical genotype isolated from *A. mellifera* in South Korea (53). IAPV and BQCV were detected only in certain farms (**Figure 1**). Interestingly, there were discrepancies between the results of HTS and PCR as shown in **Figure 1**: only two discrepancies were observed among the 96 analyses (16 viruses in six regions). CBPV was identified by PCR, whereas ABPV was detected only by HTS. This result suggests that HTS sometimes fails to detect low-abundance viral sequences or underestimates the viral load (55). In contrast, due to technical limitations such as the primer conditions, sample concentration, and amplicon quality, some viruses may not be detected by PCR (56, 57). According to **Supplementary Data 3** in **Supplementary File 1**, CBPV showed the lowest TPM value among the 11 viruses in farm 2, with an extremely low read count of viral reads, indicating relatively low viral abundance. Detecting such low-abundance viruses by PCR would require more amplification cycles, which could result in nonspecific amplification and a risk of false-positive results (58). KBV exhibits sequence homology of up to 70% with IAPV (59); however, none of the PCR and HTS results indicated the presence of KBV. Considering that we confirmed the distribution and presence of the virus using two methods, PCR and HTS, further studies using small RNA sequencing to detect viral siRNAs within mites are required to confirm whether honey bee-pathogenic viruses replicate within *V. destructor* (24).

We identified several *Varroa destructor* viruses in this study, including VDV2, VDV3, VDV4, VDV5, VDV9, and VOV1, of which four viruses: VDV2, VDV3, VDV5, and VDV9, were detected in all six regions, whereas VDV4 was detected in three regions. In a previous study, Eliash et al. (11) investigated the distribution of these viruses in 66 V*. destructor* samples and detected VDV2 in all SRA samples, with a 100% prevalence. VDV5 was detected in 67% of samples, whereas VDV3 and VDV4 had lower prevalence rates of 35% and 17%, respectively (11). VDV9 was not found in the analysis because its genome sequence was unknown at that time (24). Consistent with these previous findings, when we evaluated the abundance of various VDVs in *Varroa* mites and quantitatively compared their abundances within the samples, our results confirmed the presence of VDV2 in all six regions, with the lowest prevalence observed for VDV4. Moreover, we conducted a quantitative comparison of VDVs within each sample using TPM values to determine the relative abundance (**Figure 2**, **Supplementary Data 3** in **Supplementary File 1**). In most samples, VDV3 showed the highest abundance, followed by VDV5, VDV9, and VDV2, except in farm 6. Irrespective of whether DWV was dominant, the relative abundance of VDV3 was consistently highest across the samples. A similar study in New Zealand investigated the relative abundance of VDVs in 27 bee hives with *V. destructor* infestation and reported a distribution pattern of VDV2 > VDV9 > VDV3 > VDV5 (34). Unlike the results from New Zealand, our study showed a relatively low abundance of VDV2 in all regions. In another study, Lester et al. (34) found a negative relationship between VDV2 and DWV, where a high abundance of VDV2 in *V. destructor* mites was associated with a reduced abundance of DWV in the mites. In our study, the hypothesis did not fit that farm 2, which had an extremely low abundance of DWV, exhibited VDV3 as the predominant virus, whereas VDV2 was relatively less abundant. This result suggests a lack of correlation between the abundances of VDV2 and DWV, thus supporting the results of Herrero et al. (2019) (60), who also found no significant associations between the two viruses. Various VDV strains are widely distributed within *V. destructor* populations; however, there are limited studies on their potential virulence in mite populations and the dynamics of viral transmission. Therefore, additional research is required to examine the distribution and abundance of VDVs in *V. destructor*, thereby gaining a better understanding of the interactions between viruses and their hosts.

Our analysis revealed the presence of ARV1, ARV2, HPLV34, and LLCV, whose major hosts have not been clearly identified. ARV1 was detected in all six regions. This virus has also been reported in *A. mellifera* populations in Africa, Asia, Europe, and the Pacific region (61). Phylogenetic analysis revealed that our ARV1 strain belonged to the same branch as the Chinese genetic lineages (**Figure 5**). ARV2, which shared approximately 50% nucleotide identity with ARV1, was confirmed as a distinct virus and was also detected in all regions. Nevertheless, due to the limited availability of ARV2 genome sequences in NCBI, we were unable to construct a comprehensive phylogenetic tree. Furthermore, we detected HPLV34 and LLCV, neither of which has been previously reported in *V. destructor*. HPLV34 was also previously identified in honey bees (33, 62). LLCV is a member of the genus *Ilarvirus* (family *Bromoviridae*) and contains tripartite genome segments RNA1, RNA2, and RNA3 (63). Analysis at 40× sequencing depth consistently detected LLCV RNA1 and RNA3 segments in full, whereas the RNA2 segment was not detected. Hence, we hypothesized that increasing the sequencing depth would enable us to obtain a complete set of viral genomes. We reanalyzed the same sample at 90× sequencing depth and successfully identified the previously undetected LLCV RNA2 segment (**Figure 6**). These results demonstrated that increased sequencing depth improved the accuracy of viral genome detection and the identification of genomic variations (64). Our results confirmed the presence of LLCV in *Varroa* mites; therefore, we raised the question: how do plant viruses infect *Varroa* mites? Such previously unreported sequences may derive from HTS artifacts. This phenomenon, known as index hopping, occurs when multiple samples are processed within a single sequencing lane (65). To exclude the possibility of HTS artifacts, we conducted RT-PCR to amplify the viral sequence and confirmed the presence of the virus in the RNA sample. The presence of plant viruses in *V. destructor* indicates two hypotheses: (1) contamination by pollen on the surface or inside of *V. destructor*, and (2) *V. destructor* acting as a new host for plant-associated viruses. Lee et al. (2023) (66) reported the presence of LLCV in pollen and bee bread metagenomes, supporting the hypothesis of contamination via pollen. Pollen is a major route for the transmission of plant viruses to honey bees; however, limited knowledge exists about the dynamics of cross-species transmission to *Varroa* mites via pollen (67). Regarding the second hypothesis that *V. destructor* could serve as a new host for plant-associated viruses, previous research has supported the hypothesis that tobacco ringspot virus, which replicates in honey bees, was present in *V. destructor* (68). To summarize, both hypotheses could explain the presence of plant viruses in *V. destructor*, and further studies are necessary to determine whether these viruses actively replicate within *V. destructor.*

Based on previous studies, 26 types of viruses have been identified in *Varroa* mites (11, 24). However, in our samples collected in 2022, 10 of the 26 viruses were not detected. To confirm the absence of these viruses, the sequencing reads were aligned to each reference genome as a means of resequencing. Our results showed no evidence for the presence of the 10 viruses that had been previously reported in *V. destructor*, including slow bee paralysis virus (SBPV), bee macula-like virus (BMLV), Lake Sinai virus (LSV), Apis flavivirus (AFV), Apis mellifera filamentous virus (AmFV), *Varroa* mite associated genomovirus 1 isolate VPVL_46 (VPVL 46), KBV, *Varroa* Tymo-like virus (VTLV), Apis mellifera nora virus 1 (ANV), and *Varroa* mite associated genomovirus 1 isolate VPVL_36 (VPVL 36). Although virome studies have primarily focused on honey bees due to their economic importance in agriculture (19, 33, 34), considering the potential role of *Varroa* mites as a vector for honey bee viruses, continuous monitoring of the viral composition and relative abundance of *Varroa* mites according to region and time could provide critical insights into interactions between the parasite and virus, as well as their impact on the honey bee ecosystem.

## Data Availability

The datasets presented in this study can be found in online repositories. The names of the repository/repositories and accession number(s) can be found in the article/**Supplementary Material**.
