# Supplementary material for: Comprehensive virome analysis of Varroa destructor populations in South Korea
Source: Front Insect Sci. 2026 Jan 28;6:1757017. doi: 10.3389/finsc.2026.1757017 (PMC12891166; doi:10.3389/finsc.2026.1757017)
Supplement: Supplementary file 1 [file SupplementaryFile1.docx]

Supplementary Material

# Supplementary Data

# Supplementary Figures and Tables

## Supplementary Figures

**List of Supplementary Tables:**

Supplementary_Table _1_

Supplementary_Table _2_

Supplementary_Table _3

Supplementary_Table _4

Supplementary_Table _5

**2.2 Supplementary Figures**

Supplementary_Figure _1

Supplementary_Figure _2

| **No** | **Farm** | **Location** | **GPS** |
| --- | --- | --- | --- |
| 1 | Farm 1 | 117, Deok-ri, Bongdam-eup, Hwaseong-si, Gyeonggi-do | 37°10′11.3″N 126°56′35.8″E |
| 2 | Farm 2 | 2-5, Wonam-ri, Namsa-eup, Cheoin-gu, Yongin-si, Gyeonggi-do | 37°05′12.3″N 127°10′46.1″E |
| 3 | Farm 3 | 546-1 Seohwang-ri, Bukcheon-myeon, Hadong-gun, Gyeongsangnam-do | 35°7′55.92″N 127°52′50.88″E |
| 4 | Farm 4 | 1112-2, Sajeong-ri, Eumseong-eup, Eumseong-gun, Chungcheongbuk-do | 36°58′12.9″N 127°37′26.0″E |
| 5 | Farm 5 | 10, Geumseong-ro 23beon-gil, Gangneung-si, Gangwon-do | 37°41′37.9″N 128°53′37.7″E |
| 6 | Farm 6 | **1425, Susan-ri, Aewol-eup, Jeju-si, Jeju-do** | 33°27′27.53″N 126°23′12.73″E |

**Supplementary Table 1.** Geographical distribution of *V. destructor* collection sites across six farms in South Korea. Farms 1 and 2 are located in Gyeonggi-do; Farm 3 Gangwon-do; Farm 4 in Chungcheongbuk-do; Farm 5 in Gyeongsangnam-do; and Farm 6 in Jeju-do.

| **Sample** | **Number of samples** | **Total number of bases (bp)** | **Total reads** | **GC (%)** | **AT (%)** | **Q20 (%)** | **Q30 (%)** |
| --- | --- | --- | --- | --- | --- | --- | --- |
| Farm 1 | 20 | 10,469,416,182 | 69,333,882 | 40 | 59.1 | 98.5 | 96 |
| Farm 2-1* | 10 | 11,352,262,446 | 75,180,546 | 46 | 53.9 | 98 | 95.1 |
| Farm 2-2* | 10 | 25,372,508,256 | 168,029,856 | 46 | 54 | 98 | 94.7 |
| Farm 3 | 20 | 13,358,091,180 | 88,464,180 | 41 | 58.8 | 98.5 | 96.1 |
| Farm 4 | 10 | 10,906,271,564 | 72,226,964 | 41 | 59 | 98.5 | 96.1 |
| Farm 5 | 10 | 11,922,817,456 | 78,959,056 | 40 | 59 | 98.6 | 96.4 |
| Farm 6 | 10 | 11,863,772,530 | 78,568,030 | 44 | 55 | 98.1 | 95.2 |

**Supplementary Table 2**. Summary of sequencing preprocessing statistics

The details in each column are based on the information provided by sequencing analysis. The red mark indicates that farm 2 samples were analyzed at two different sequencing depths.

| **Type** | **Family** | **Virus**  **name** | **Virus**  **abbreviation** | **Virus detection in this study** |
| --- | --- | --- | --- | --- |
| ssRNA (+) | Iflaviridae | Deformed wing virus | DWV | + |
|  |  | Slow bee paralysis virus | SBPV | - |
|  |  | Sacbrood virus | SBV | + |
|  |  | *Varroa destructor* virus 2 | VDV2 | + |
|  | Dicistroviridae | Israel acute paralysis virus | IAPV | + |
|  |  | Kashmir bee virus | KBV | - |
|  |  | Acute bee paralysis virus | ABPV | + |
|  |  | Black queen cell virus | BQCV | + |
|  | Tymoviridae | Bee macula-like virus | BMLV | - |
|  |  | *Varroa* Tymo-like virus | VTLV | - |
|  | Flaviviridae | Apis flavivirus | AFV | - |
|  | Unclassified ssRNA (+) | Lake Sinai virus | LSV | - |
|  |  | *Varroa destructor* virus 3 | VDV3 | + |
|  |  | *Varroa destructor* virus 5 | VDV5 | + |
|  |  | *Varroa destructor* virus 9 | VDV9 | + |
|  |  | Chronic bee paralysis virus | CBPV | + |
| ssRNA(-) | Rhabdoviridae | Apis rhabdovirus 1 | ARV1 | + |
|  |  | Apis rhabdovirus 2 | ARV2 | + |
|  | Orthomyxoviridae | *Varroa orthomyxovirus-1* | VOV1 | + |
|  | Unclassified ssRNA (–) | *Varroa destructor* virus 4 | VDV4 | + |
| DNA | Genomoviridae | *Varroa* mite associated genomovirus 1 isolate VPVL_36 | VPVL36 | - |
|  | Unclassified dsDNA | Apis mellifera Filamentous virus | AmFV | - |
|  | Unclassified ssDNA | *Varroa* mite associated virus 1 isolate VPVL_46 | VPVL46 | - |

**Supplementary Table 3. List of *V. destructor*-infected viruses**

List of viruses reported in previous studies. Viruses detected in this study are indicated with a plus sign (+), while the symbol “-” indicates its absence.

| **Accession Number** | **Virus** | **Farm 1** | **Farm 2** | **Farm 3** | **Farm 4** | **Farm 5** | **Farm 6** |
| --- | --- | --- | --- | --- | --- | --- | --- |
| AY292384.1 | Deformed wing virus | **994,854** | **97** | **997,560** | **989,417** | **760,087** | **898,824** |
| AF092924 | Sacbrood virus | **162** |  |  |  |  |  |
| OR496458 | Israeli acute paralysis virus |  |  | **2** | **843** |  |  |
| NC003784 | Black queen cell virus | **780** |  |  |  |  |  |
| OM744312 | Acute bee paralysis virus |  |  |  |  |  |  |
| OR584326 | Chronic bee paralysis virus |  | **72** |  |  |  |  |
| NC040601 | *Varroa destructor* virus 2 | **66** | **7,965** | **45** | **110** | **156** | **3,650** |
| KX578272 | *Varroa destructor* virus 3 | **1,857** | **483,819** | **986** | **5,093** | **2,026** | **6,867** |
| MK032464 | *Varroa destructor* virus 4 | **4** | **716** |  | **3** |  | **295** |
| MK795519 | *Varroa destructor* virus 5 | **620** | **106,891** | **495** | **663** | **346** | **39,007** |
| OR224321 | *Varroa destructor* virus 9 | **1,634** | **394,742** | **856** | **3,736** | **1,974** | **50,111** |
| OL803863 | *Varroa orthomyxovirus* | **4** | **2,969** | **52** | **115** | **212** |  |
| OR496404 | Apis rhabdovirus 1 | **6** | **837** | **2** | **8** | **31** | **801** |
| OR496500 | Apis rhabdovirus 2 | **14** | **1,195** | **2** | **10** | **14** | **444** |
| NC025477/ NC025478/  NC025481 | Lilac leaf chlorosis virus |  | **695** |  |  |  |  |
| ON648754 | Hubei partiti-like virus 34 |  |  |  |  | **235,153** |  |

**Supplementary Figure 1. Relative abundance of 16 *V. destructor* viruses**

A heatmap revealing the relative abundances of the 16 viruses in the *V. destructor* samples collected from six different farms. Color intensity represents the values of viral relative abundance, calculated as transcripts per million (TPM). Darker colors indicate higher relative abundance. White boxes denote the absence of viral transcripts.

| **Virus name** | **Abbreviation** | **F/R** | **Primer sequence (5′ → 3′)** | **Amplicon size (bp)** | **Reference** |
| --- | --- | --- | --- | --- | --- |
| Deformed wing virus A | DWV | F | TCATCTTCAACTCGGCTTTCTA | 480 | (Lee et al., 2005) |
|  |  | R | CGAATCATTTTCACGGGACG |  |  |
| Sacbrood virus | SBV | F | ACCAACCGATTCCTCAGTAG | 487 | (Grabensteiner et al., 2001) |
|  |  | R | CCTTGGAACTCTGCTGTGTA |  |  |
| Israeli acute paralysis virus | IAPV | F | GGTGTCGAGGAGGACTTGA | 610 | (Moon et al., 2024) |
|  |  | R | CTGTGCGTATCATCCATGTTC |  |  |
| Chronic bee paralysis virus | CBPV | F | GGAAGTCATCCGTAGATCTG | 479 | (Moon et al., 2024) |
|  |  | R | GGCGGTGGTCTCGTTCTT |  |  |
| Black queen cell virus | BQCV | F | GGACGAAAGGAAGCCTAAAC | 424 | (Nielsen et al., 2008) |
|  |  | R | ACTAGGAAGAGACTTGCACC |  |  |
| Voltage-sensitive sodium channel | VSSC | F | CTAGCCAAGTCATGGCCAAC | 146 | (Lee et al., 2023) |
|  |  | R | TTGTCGAGATAGTTCTTGCCG |  |  |
| Acute bee paralysis virus | ABPV | F | ACAGCCAACCTTCTGGTAATC | 489 | This study |
|  |  | R | GCATGGAAAGCTCCATAATC |  |  |
| *Varroa destructor* virus 2 | VDV2 | F | TATGGGCGTGGAGGGAGTTA | 228 | This study |
|  |  | R | GCAAACACCGTCAGTGGAAC |  |  |
| *Varroa destructor* virus 3 | VDV3 | F | CCACGCCCGGTTAGCTTTGAG | 578 | This study |
|  |  | R | CAGCCATCATCGGTCGTCTAA |  |  |
| *Varroa destructor* virus 4 | VDV4 | F | TGGGGATGCCGCTACATTTT | 437 | This study |
|  |  | R | TCACCTCCGTACGGGATTCT |  |  |
| *Varroa destructor* virus 5 | VDV5 | F | TAAGAGCAAAGGCCCTATCCCT | 308 | This study |
|  |  | R | ATCCAAAGGTCCCTCATGCAAT |  |  |
| *Varroa destructor* virus 9 | VDV9 | F | GTTACATGCCGTTGCGGTTT | 413 | This study |
|  |  | R | GCTAACAGGGAAGGCCAGTT |  |  |
| *Varroa orthomyxovirus* | VOV1 | F | GTACAAGCACCTGCATTCGTG | 514 | (Kwon et al., 2023) |
|  |  | R | GGCCTGTAGAGAGGACCAGA |  |  |
| Apis rhabdovirus1 | ARV1 | F | GGAACGGTGACTCCTTCGTT | 573 | This study |
|  |  | R | GGGGGAAATATGGGTGCCAA |  |  |
| Apis rhabdovirus2 | ARV2 | F | TCCTATCCCCGCCAAGAAGA | 330 | This study |
|  |  | R | ATAGGGGTGCCCAAAAGCTC |  |  |
| Hubei paritit-like virus 34 | HPLV34 | F | TGCGTGTGTAAAAATCACTGGA | 457 | (Kwon et al., 2023) |
|  |  | R | CGGTGGAAGTGTGGAGAGAC |  |  |
| Lilac leaf chlorosis virus | LLCV RNA1 | F | GCATTGACGAGGGAGGCTAA | 281 | This study |
|  |  | R | GCGTGCTGAAGGAAACACTC |  |  |
| Lilac leaf chlorosis virus | LLCV RNA2 | F | TGAAAGACCGTTAGCTGCGA | 520 | This study |
|  |  | R | TCGGTCCAAGGGTTTTACGG |  |  |
| Lilac leaf chlorosis virus | LLCV RNA3 | F | GAAACCGGTGCAGTACCTGA | 543 | This study |
|  |  | R | CGGTCCAAGAAGTCGGAACA |  |  |

**Supplementary Table 5.** List of primers used in this study.


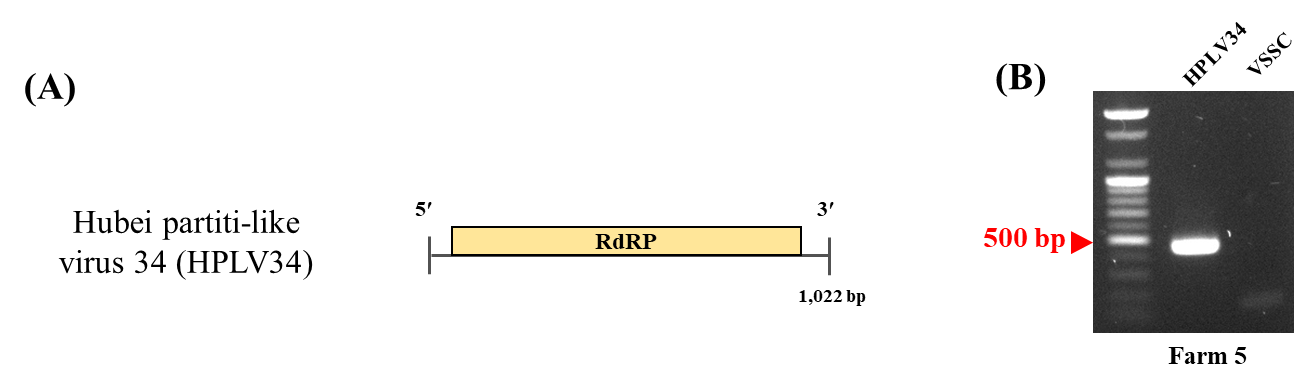
 **Supplementary Figure 2.**

(A) Genome sequence of Hubei partiti-like virus 34 (HPLV34) in *V. destructor cells.* The viral genome contains an RNA-dependent RNA polymerase (RdRP).

(B) RT-PCR analysis of HPLV34.

Gel electrophoresis confirmed the presence of HPLV34 using RT-PCR. The positive control is represented by the voltage-sensitive sodium channel (VSSC).
